# CHANGES IN ACCELEROMETER MEASURED MOVEMENT BEHAVIORS OVER RETIREMENT: A COMPOSITIONAL DATA APPROACH

**DOI:** 10.1093/geroni/igad104.0130

**Published:** 2023-12-21

**Authors:** Lawrence Sacco, Hugo Westerlund

**Affiliations:** Stockholm University, Stockholm, Stockholms Lan, Sweden; Stockholm University, Stockholm, Stockholms Lan, Sweden

## Abstract

Retirement is a significant life transition where individuals change their daily patterns of movement behaviors (i.e. physical activity and sedentary behavior). However, evidence accounting for how individuals re-allocate time from one type of activity to another is lacking, considering that engagement in different activities is co-dependent as time not spent in a given type of activity will be spent in another one. The objective of this paper is to evaluate how relative time spent in movement behaviors (light physical activity, moderate-vigorous physical activity and sedentariness) and sleep change over the retirement transition. Furthermore, the aim is to assess how movement behaviors change differently between genders, manual Vs non-manual work and weekend Vs weekdays. Data was collected from 114 participants (47 men and 67 women; ages: 60 to 73) from the Swedish Retirement Study (SRS) at three timepoints: 6 months before retirement and, 6 and 18 months after retirement. Time-use data on movement behaviors and sleep was collected over a week-long period through thigh-worn accelerometers and wrist-worn actigraphs. Compositional data analysis (CoDA) was used to account for the compositional and finite nature of 24 hour time-use data. Results indicate that small changes in allocation of time between movement behaviors occur during retirement. Changes indicate that after retirement weekdays shows patterns of activity that resemble more patterns typical of pre-retirement weekends. Findings are discussed in relation to implications for physical activity guidelines and the influence of retirement on health.

